# Early assessment of diabetes care target attainment in Mexico’s federalized public health system: alignment with Global Diabetes Compact 2030

**DOI:** 10.3389/fcdhc.2026.1837250

**Published:** 2026-06-15

**Authors:** Rubén Silva-Tinoco, Alejandro Avalos-Bracho, Vladimir Martínez-de la Cruz, Gabriel Gutiérrez-Morales, Arturo González-Ledesma, Arlette Saavedra-Romero, María Fernanda Bernal-Ceballos

**Affiliations:** 1 Unidad de Atención a la Salud, Servicios Públicos de Salud del Instituto Mexicano del Seguro Social para el Bienestar, Mexico City, Mexico; 2 Coordinación de Unidades de Primer Nivel de la Unidad de Atención a la Salud, Servicios Públicos de Salud del Instituto Mexicano del Seguro Social para el Bienestar, Mexico City, Mexico; 3 Coordinación de Supervisión de la Unidad de Atención a la Salud, Servicios Públicos de Salud del Instituto Mexicano del Seguro Social para el Bienestar, Mexico City, Mexico

**Keywords:** diabetes care, diabetes complications, disease burden, health information system (HIS), health policy, healthcare quality, healthcare system, primary care

## Abstract

**Background:**

Robust system-level monitoring tools are essential to assess the performance of diabetes care within healthcare systems. We report an early assessment from an integrated monitoring platform in Mexico’s emerging federalized public health system, focusing on indicators aligned with the World Health Organization Global Diabetes Compact 2030 targets.

**Methods:**

We conducted a descriptive health services research analysis using routinely collected clinical data from a national monitoring platform that enables longitudinal and subnational analyses. Key system-level indicators included attainment of glycemic control, blood pressure control, and statin use.

**Results:**

From 2023 to September 2025, data from 544,978 individuals across participating states were registered in the platform, of whom 316,947 with a recent diabetes care record were included in the analysis. Among individuals with a recent diabetes care record, 59.3% achieved glycemic control, 76.6% achieved blood pressure control, and 30.2% were receiving statin therapy. Marked geographic heterogeneity was observed across states, with glycemic control ranging from 49% to 70%, blood pressure control from 65.8% to 82.4%, and statin use from 15.3% to 53%. Women showed higher attainment of blood pressure control and statin use, whereas older individuals demonstrated higher glycemic control than younger age groups.

**Conclusions:**

Routine clinical data provides valuable system-level insights into progress toward diabetes care targets. Early findings from Mexico’s emerging federalized public health system reveal substantial gaps and territorial variation in key indicators, highlighting priority areas for strengthening diabetes care in primary healthcare.

## 1 Introduction

Diabetes represents one of the major challenges for health systems worldwide, contributing substantially to morbidity, mortality, and healthcare expenditures (1). The burden of diabetes is particularly high in low- and middle-income countries, where health systems face increasing demand for chronic disease management. In Mexico, diabetes is a leading cause of premature mortality and disability, with adult prevalence estimates exceeding 18% and a substantial impact on clinical outcomes and health system utilisation (2–4).

Effective management of diabetes requires sustained delivery of evidence-based interventions to attain relevant clinical targets, including glycemic control, blood pressure management, and reduction of the risk of diabetes-related complications (5–8). However, despite advances in diabetes care over the past two decades, suboptimal management of diabetes remains a major global challenge, particularly in low- and middle-income countries (9). Moreover, many health systems still struggle to consistently monitor the quality of diabetes care and track progress toward key clinical targets at the population level (10).

Robust health information systems play a central role in supporting improvements in the management of chronic conditions (11, 12). Routine clinical data can provide valuable insights into health system performance, enabling the identification of gaps in care delivery and informing targeted interventions. When systematically analysed, these data can support continuous monitoring of care processes and outcomes, helping health systems translate information generated during clinical practice into opportunities for improvement (13–15).

In Mexico, the recent federalization of public health services has led to the consolidation of healthcare delivery for populations without social security coverage, representing approximately 50 million people, under the IMSS-Bienestar system (16). This model was designed to expand access to free healthcare, strengthen primary care services, and standardise the quality of care while reducing longstanding regional inequities across participating states (17). Since 2023, 23 of 32 states have adhered to this federalized system, which oversees the largest network of primary healthcare facilities in the country, comprising 9,610 units. The scale and territorial diversity of this network create both opportunities and challenges for monitoring the quality of diabetes care, highlighting the need for tools capable of analysing routine clinical data across jurisdictions and over time.

At the global level, the Global Diabetes Compact launched by the World Health Organization (WHO) in 2021 established a set of targets for 2030 aimed at improving health outcomes for people living with diabetes through enhanced detection, treatment, and cardiovascular risk management (18, 19). The Global Diabetes Compact provides a pragmatic monitoring framework for diabetes care performance, aligned with established approaches to quality measurement and health system improvement. For individuals with diagnosed diabetes, the proposed 2030 targets include: (1) 80% achieving glycemic control (HbAc1 < 8%), (2) 80% achieving blood pressure control (<140/90 mmHg), and (3) 60% of individuals aged 40 years or older receiving statin therapy. These indicators were selected given their strong association with reductions in diabetes-related complications and cardiovascular mortality, as well as their feasibility for monitoring using routinely collected clinical data (18). Together, these targets provide an important framework for monitoring progress in diabetes care and guiding health system strengthening efforts (20). In this context, the present study reports early system-level monitoring findings derived from an integrated monitoring platform using routinely collected clinical data, focusing on indicators aligned with the Global Diabetes Compact 2030 targets within Mexico’s emerging federalized public health system.

## 2 Methods

### 2.1 Study design and data source

A descriptive health services research analysis was conducted using routinely collected clinical data obtained from the Integrated Monitoring Platform for Actionable Diabetes Care (IMPACT-Diabetes Care), designed to support system-level evaluation of diabetes care within the IMSS-Bienestar public health system. The platform consolidates data from the national chronic diseases routine collected data information system (*Sistema de Información en Crónicas*, SIC), which captures standardized clinical information from primary healthcare facilities. Data are entered either directly by healthcare providers during clinical encounters or subsequently recorded from structured clinical follow-up forms used in routine care.

Through structured data integration and processing, the platform enables the generation of actionable indicators for system-level monitoring, facilitating longitudinal and subnational analyses of diabetes care performance across the 23 participating states. The platform focuses on individuals with recent documented contact with care, enabling the assessment of clinical processes and outcomes among those actively engaged in the health system. Data is updated and analyzed monthly, enabling regular review of health system performance without adding reporting burden at the facility level. A schematic overview of the reproducible data pipeline, including data capture, privacy safeguards, quality-control procedures, cohort construction, and monitoring outputs, is presented in **Supplementary Figure 1**.

The study was approved by the IMSS-Bienestar Institutional Ethics Committee (IB-2026-00007) in accordance with applicable regulations. The institutional review boards granted a waiver of written informed consent, as the study involved secondary analysis of routinely collected and anonymised clinical data obtained during standard care.

### 2.2 Study population

The analysis included adults (≥ 18 years) with a documented diagnosis of diabetes receiving care within the IMSS-Bienestar primary healthcare network. In the analytic dataset, diabetes was operationalized by a completed year-of-diabetes-diagnosis field in the SIC platform, indicating a documented clinical diagnosis recorded during routine care. Records from January 2023 to September 2025 were considered. For the purposes of system-level monitoring, the analytic population was defined as individuals with at least one recent clinical record related to diabetes care, operationalized as any documented encounter within the six months preceding September 2025. This definition reflects the population actively engaged in care, for whom clinical processes and outcomes can be meaningfully assessed using routinely collected data. Individuals without a clinical encounter within this period were excluded, as they do not have contemporaneous clinical or laboratory data and therefore cannot contribute to the estimation of current care indicators. No *a priori* sample size calculation or power analysis was performed, as the study included all eligible records available in the institutional clinical database during the predefined study period.

### 2.3 Statistical analysis

We conducted a descriptive analysis of routinely collected clinical data, estimating proportions of individuals meeting predefined diabetes care targets. For each anchor month, estimates were stratified by sex, age group, and state, when applicable.

The analytical framework was designed to align routinely collected real-world data with the WHO Global Diabetes Compact targets, enabling direct comparison with global benchmarks. Key diabetes care indicators included glycemic control, defined as <8.0%; blood pressure control, defined as <140/90 mmHg; and statin use among individuals aged ≥40 years. Indicator attainment was calculated as the proportion of individuals meeting each target among those with available data within the monitoring period, and should therefore be interpreted as conditional on measurement availability.

Indicator-specific denominators were defined based on data availability within the monitoring period. Glycemic control and lipid-related indicators were calculated among individuals with available laboratory measurements, while blood pressure control was assessed among those with recorded measurements within the defined time window. For clinical indicators, the latest recorded value available within the predefined six-month monitoring window was used for each individual, including HbA1c and blood pressure measurements. Statin use was evaluated among individuals aged ≥40 years with available prescription records. For each estimate, 95% confidence intervals were calculated to provide measures of precision.

Temporal trends in population coverage were assessed using monthly aggregated counts of individuals receiving diabetes care. Given the descriptive and system-level monitoring nature of the study, no formal hypothesis testing or multivariable analyses were performed, and comparisons across subgroups are presented descriptively. Individuals were nested within healthcare facilities and states, which may influence the precision of estimates; this was considered in the interpretation of results rather than through formal modeling.

Data processing and validation were conducted in parallel using R version 4.4.2 (R Foundation for Statistical Computing, Vienna, Austria) and Python version 3.12 (Python Software Foundation, Wilmington, Delaware, USA).

## 3 Results

**Figure 1:**
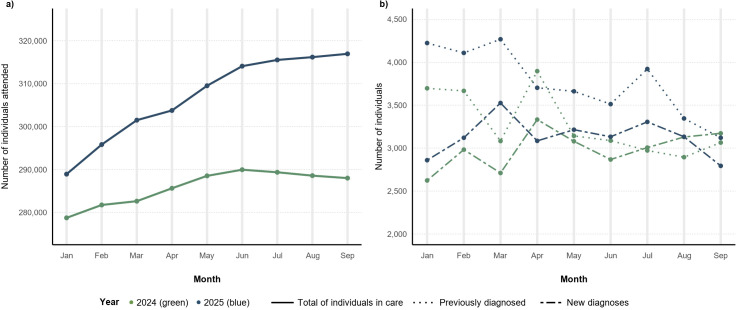
Trends in population coverage for diabetes care including total individuals attended **(A)**, newly diagnosed individuals initiating care, and previously diagnosed individuals registered for the first time **(B)**. Data source: Sistema de Información en Crónicos (SIC).

A total of 544,978 individuals with diabetes attended and captured in the SIC platform between 2023 and 2025. The derivation of the analytic sample is shown in **Supplementary Figure 2**. Trends in the number of individuals receiving diabetes care over time, including new diagnoses and individuals initiating care within the healthcare system, are shown in **Figures 1A, B** respectively. Over the study period, the number of individuals who received care for diabetes was consistently higher in 2025 compared with 2024. In 2024, the number of individuals attending remained relatively stable and increased modestly by ≈3.3% (n=278,762 in January vs n=288,012 in September). In contrast, 2025 showed a progressive increase of ≈9.7% over the same period of time (n=288,939 in January vs n=316,947 in September). Overall, by September, the number of individuals receiving care in 2025 was 13.6% higher than in January 2024, indicating an expansion in the population receiving diabetes care.

Characteristics of individuals with a recent clinical record during the monitoring period are presented in **Supplementary Table 1**. Most of the individuals receiving care were women, representing 71.7% (n=227,466). The population was predominantly older, with 49.8% aged ≥60 years, 44.1% aged 40–59 years, and only 5.9% younger than 40 years. The mean BMI was 29.6 ± 5.5 kg/m^2^ and regarding diabetes duration, 37% had lived with diabetes <5 years, whereas approximately 40% for more than 10 years. Chronic comorbidities were common, particularly hypertension (48.3%) and obesity (37.4%), while cardiovascular disease was reported in only 2.2% of patients. HbA1c measurements were available for 60.5% of individuals, among whom 42.6% achieved the strict glycemic target (<7.0%). Similarly, total cholesterol measurements were recorded in 60.4% of individuals, and among those with available measurements, 66.5% achieved the target of <200 mg/dL. Comparisons of individuals with and without available HbA1c or cholesterol measurements showed some differences in demographic and clinical characteristics, supporting cautious interpretation of laboratory-based indicators; detailed results are presented in **Supplementary Tables 2**, **3**.

**Figure 2:**
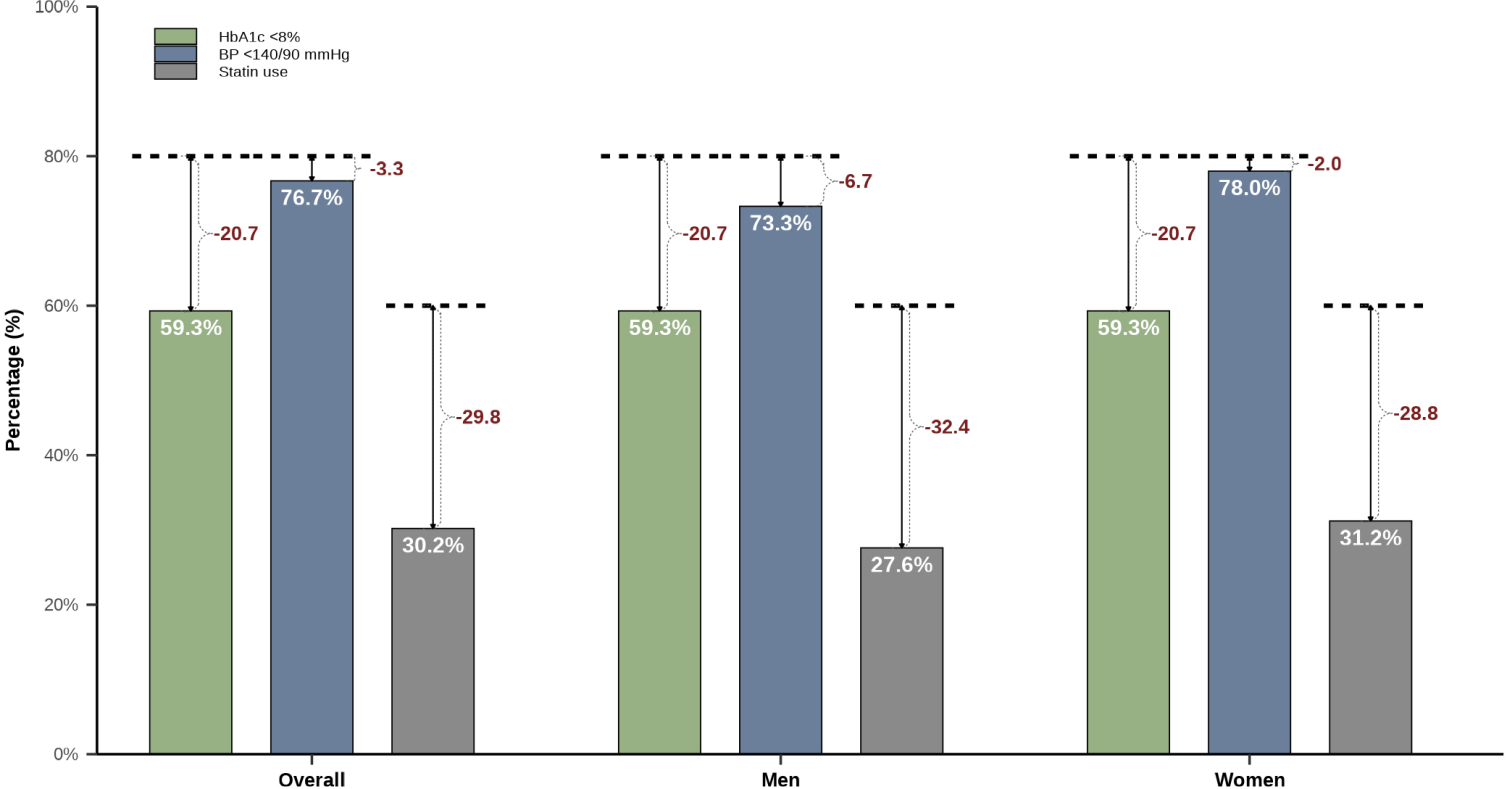
Attainment of key diabetes care targets and gap to Global Diabetes Compact 2030 targets, overall and by sex, among individuals receiving diabetes-related care in the IMSS-Bienestar health system (n = 316,947), based on the September 2025 assessment using a 6-month rolling window. Bars represent observed attainment of glycemic control, blood pressure control, and statin use, while dashed lines indicate the corresponding Global Diabetes Compact 2030 targets. Data source: *Sistema de Información en Crónicas* (SIC). HbA1c: glycated hemoglobin; BP: blood pressure. Dashed lines represent the WHO Global Diabetes Compact targets: 80% for HbA1c <8% and BP <140/90 mmHg, and 60% for statin use. Red numbers indicate the gap in percentage points (pp) between the observed attainment and the WHO Global Diabetes Compact target. Estimates are conditional on measurement availability; denominators vary by indicator. HbA1c <8% was calculated among individuals with a recorded HbA1c value, blood pressure <140/90 mmHg among those with a recorded measurement, and statin use among individuals aged ≥40 years with a recorded prescription.

The overall attainment of the three key diabetes care targets is shown in **Figure 2**. Estimates are presented with 95% confidence intervals in **Supplementary Table 4**. Blood pressure control showed the highest level of attainment among the evaluated indicators and was closest to the Global Diabetes Compact target (76.6%). In contrast, glycemic control remained below the target, while statin use showed the lowest overall attainment (59.3% and 30.2%, respectively). When stratified by sex, similar patterns were observed in both groups. Compared with men, women demonstrated higher attainment of blood pressure control (78.0% vs 73.3%) and greater statin use (31.2% vs 27.6%), while glycemic control was similar between groups.

**Figure 3:**
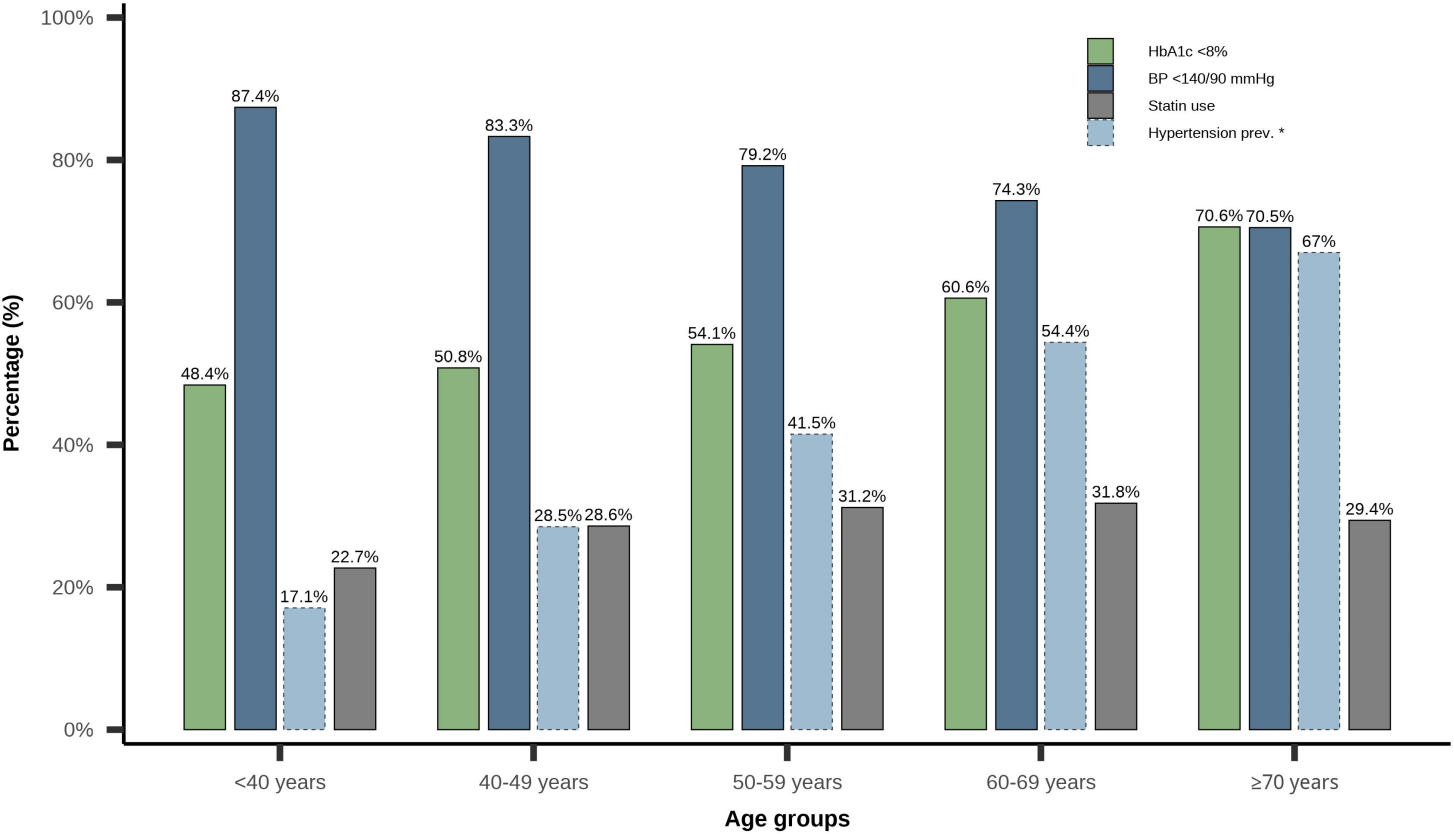
Attainment of diabetes care targets and hypertension prevalence by age group, among individuals receiving diabetes-related care in the IMSS-Bienestar health system (n = 316,947), based on the September 2025 assessment using a 6-month rolling window. Data source: *Sistema de Información en Crónicas* (SIC). HbA1c: glycated hemoglobin; BP: blood pressure. White dashed lines within the BP control bars represent the prevalence of hypertension within each age group and are shown for contextual interpretation; they do not represent treatment targets. Estimates are conditional on measurement availability; denominators vary by indicator. HbA1c <8% was calculated among individuals with a recorded HbA1c value, blood pressure <140/90 mmHg among those with a recorded measurement, and statin use among individuals aged ≥40 years with a recorded prescription.

Attainment of diabetes care targets and hypertension prevalence by age group are shown in **Figure 3**. After stratification by age group, the proportion of individuals achieving glycemic control increased progressively across age groups, with the highest attainment observed among those aged ≥70 years (70.6%). Similarly, statin use increased steadily with age, reaching the highest level among individuals aged 60–69 years (31.8%). In contrast, the proportion of individuals achieving blood pressure control declined across age groups, decreasing from 87.4% among those aged <40 years to 70.5% among those aged ≥70 years. The prevalence of hypertension increased markedly with age, from 17.1% (2,773 of 16,174) among individuals aged <40 years to 28.5% (11,959 of 41,935) among those aged 40–49 years, 41.5% (36,931 of 89,081) among those aged 50–59 years, 54.4% (52,533 of 96,480) among individuals aged 60–69 years, and 67.0% (49,073 of 73,252) among those aged ≥70 years.

Consistent with these patterns, the most frequent combination of target attainment was the simultaneous achievement of glycemic and blood pressure control (45.9%). This was followed by the combination of blood pressure control and statin use (22.9%), and glycemic control and statin use (21.8%). In contrast, the simultaneous attainment of all three indicators was observed in only 16.7% of individuals (**Supplementary Figure 3**). Compared with individuals without recent follow-up, those with recent follow-up were more frequently women and older adults, and showed modestly better glycemic and blood pressure control, whereas several other clinical characteristics were broadly similar (**Supplementary Table 5**).

**Figure 4:**
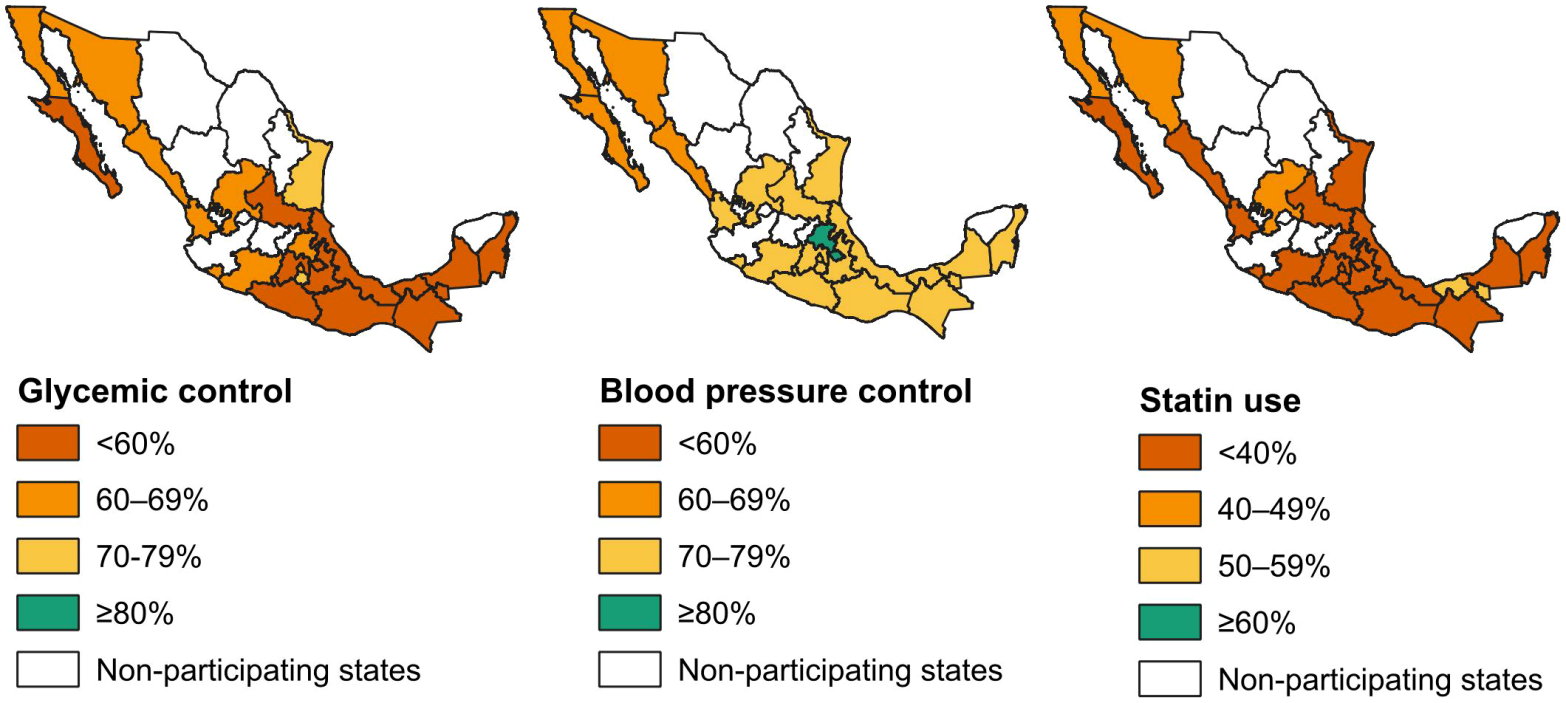
State-level heatmap of diabetes care targets attainment (glycemic control, blood pressure control, and statin use). Data source: *Sistema de Información en Crónicas* (SIC). Estimates are conditional on measurement availability; denominators vary by indicator. Glycemic control was calculated among individuals with a recorded HbA1c value, blood pressure control among those with a recorded measurement, and statin use among individuals aged ≥40 years with a recorded prescription.

Moreover, marked geographic heterogeneity in the attainment of diabetes care indicators was observed across participating states (**Figure 4**). The proportion of individuals achieving each indicator was categorised according to levels of attainment. Glycemic control showed substantial variability across the 23 participating states (12 states <60%; 9 states 60–69%; and 2 states 70–79%), followed by statin use (18 states <40%; 3 states 40–49%; 1 state 50–59%; and 0 states ≥60%). In contrast, blood pressure control showed a more homogeneous distribution across states (4 states 60–69%; 17 states 70–79%; and 2 states ≥80%).

## 4 Discussion

To our knowledge, this is among the first large-scale studies to apply the Global Diabetes Compact indicators using routinely collected clinical data to assess diabetes care at the population level within a public health system. Our findings show that, within an emerging federalized healthcare system in Mexico, blood pressure control is approaching the 2030 target, while substantial gaps persist in glycemic control and, particularly, in statin use. The implementation of an integrated monitoring platform enabled the identification of important variations in performance across states, as well as differences by sex and age groups, highlighting priority areas for improvement. These findings provide a baseline assessment of current performance and establish a foundation to track progress over time, supporting the design and implementation of strategies to improve health outcomes for people with diabetes in this evolving health system. Future repeated analyses using the monitoring platform will be important to assess temporal changes in quality-of-care indicators, progress toward care targets, and changes in regional gaps as the system continues to mature.

The observed patterns of care should be interpreted within the context of a health system undergoing rapid expansion and consolidation. The increase in the number of individuals receiving care over time reflects improved access and service coverage within the federalized model. However, the persistence of gaps in key indicators underscores ongoing challenges in achieving high-quality diabetes care. Given the chronic and progressive nature of diabetes, current levels of control are likely influenced not only by previous care delivery but also by cumulative exposures over time, including long-standing social and structural determinants of health. Disparities in chronic disease outcomes in Mexico have been widely associated with historical social disadvantage, including limited access to healthcare, lower socioeconomic status, reduced educational opportunities and lack of social security, characteristics that are frequently observed in the population attended in this healthcare system (21, 22). Consistent with this context, lower rates of glycemic control and effective coverage among individuals without social security in Mexico have been reported prior to the current healthcare model (23, 24). These contextual factors may contribute to the heterogeneity observed across regions, emphasizing the importance of monitoring approaches that are sensitive to local conditions and can inform targeted health system responses. Regional differences are likely influenced by variation in service capacity, laboratory availability, workforce distribution, local implementation conditions, population characteristics, and broader social determinants of health. The observed inter-state heterogeneity may help inform operational planning, resource prioritization, and targeted quality-improvement efforts within the health system. Understanding the determinants of these geographic differences represents an important priority for future research as the monitoring platform continues to mature. In this setting, our findings represent an early assessment of system performance, reflecting both the initial phase of implementation of a new care model and the structural challenges inherent to delivering chronic disease care in vulnerable populations. Accordingly, the findings should be interpreted as reflecting the population served by IMSS-Bienestar rather than the national population with diabetes, and direct comparisons with nationally representative surveys should be made cautiously given differences in sampling design, population composition, data sources, and indicator ascertainment.

The differential performance observed across indicators provides important insights into the organization of diabetes care within the healthcare system. Blood pressure control showed the highest level of attainment and the most consistent performance across states, while glycemic control remained below target levels, reflecting the greater complexity of achieving sustained metabolic control in routine practice. Statin use, however, emerged as the indicator with the lowest attainment, pointing to a substantial gap in cardiovascular risk management among individuals with diabetes. This finding is particularly relevant given the well-established role of statins in reducing cardiovascular morbidity and mortality, despite their relatively low cost and ease of prescription (25, 26). Within the context of the Global Diabetes Compact framework, this indicator should be interpreted as a pragmatic system-level measure of recorded statin use among adults aged ≥40 years, rather than an assessment of individualized prescribing appropriateness. Such simplified metrics may be particularly useful for large-scale monitoring across heterogeneous health systems, including settings where detailed cardiovascular risk stratification may not be consistently available.

We also observed lower engagement of male individuals in care, along with lower attainment of blood pressure control and statin use compared with females. This pattern may provide insight into the higher burden of premature mortality observed among men in Mexico, as reported in the Global Burden of Disease Study (3, 26). Additionally, the slightly lower proportion of blood pressure control among older individuals should be interpreted in the context of the higher prevalence of hypertension in this group, likely reflecting increased clinical complexity. Notably, glycemic control was suboptimal among younger age groups. Given the high burden of diabetes in Mexico, including premature mortality and early onset of disability, strengthening comprehensive care among individuals with early-onset diabetes may represent a key opportunity to reduce long-term diabetes-related complications (27–29). Taken together, these findings suggest that while some components of diabetes care are being delivered with relative consistency, others require greater prioritization and system-level reinforcement, particularly across specific regions, sexes, and age groups.

When compared with the Global Diabetes Compact 2030 targets, our findings highlight clear gaps across key indicators. Blood pressure control was closest to the proposed target, with a gap of 3.4 percentage points, whereas glycemic control and statin use remain substantially below target, with gaps of 20.7 and 29.8 percentage points, respectively. These patterns are consistent with global estimates from 2021, where among individuals with diagnosed diabetes, 63.2% achieved glycemic control, 70.8% achieved blood pressure control, and 31.8% were using statins (30). However, substantial variation was observed across country income groups. In that baseline analysis, glycemic control ranged from 56.0% in lower-middle-income countries to 73.7% in high-income countries. Together, these findings underscore persistent challenges in comprehensive risk management and the need to accelerate progress toward achieving global diabetes care targets, including strategic investment in health systems, particularly in low- and middle-income countries (28, 31). The Global Diabetes Compact provides not only quantitative targets, but also a strategic framework for advancing equitable, integrated, and people-centered diabetes care systems. Achieving these targets, however, also depends on broader quality-assurance and service-delivery strategies that may vary across health systems and were beyond the scope of the present report (32–35).

These findings have important implications for health system strengthening. First, they highlight the need to prioritize comprehensive diabetes care, particularly by increasing statin use, which represents a high-impact and feasible intervention at the primary care level, as well as by strengthening the implementation of evidence-based strategies to improve glycemic control. Prior multicomponent interventions in Mexico have demonstrated the feasibility of achieving diabetes care targets in similar settings (36, 37). Strengthening access to essential diagnostic and monitoring processes, particularly HbA1c and cholesterol measurements, will also be critical, as gaps in their execution and recording may limit the adequate assessment and optimization of care targets. Second, the observed differences by sex and age group suggest that more targeted strategies are required to improve engagement with the health system, particularly among men and individuals with early-onset diabetes, who may be at increased risk of long-term adverse outcomes. Third, the marked inter-state variability observed across indicators underscores the need for context-specific strategies and differentiated implementation efforts across regions. In this context, integrated monitoring platforms based on routinely collected clinical data can play a key role not only in identifying gaps and guiding resource allocation, but also in enabling continuous longitudinal tracking of performance, thereby supporting adaptive and data-driven improvement in diabetes care delivery across heterogeneous settings, including strengthening health information systems over time (15, 38). Future development of the monitoring platform should prioritize incorporation of social and geographic equity indicators to better assess disparities in diabetes care and outcomes.

This study has several limitations. First, the analysis is based on routinely collected clinical data, which may be subject to incomplete reporting and variability in data quality across facilities and regions. Second, the use of a recent clinical record to define the study population may not fully capture all individuals living with diabetes within the health system, potentially leading to underestimation or selection bias toward individuals more actively engaged in care. Because the study was based on routinely collected data from a specific public healthcare provider, direct comparability with nationally representative surveys or other healthcare subsystems is also limited by differences in population composition, sampling design, and indicator ascertainment. Comparisons between active and inactive individuals suggest some systematic differences in care engagement and selected outcomes, supporting interpretation of the reported estimates as reflecting the population currently engaged in care. Third, the indicators analysed reflect recorded clinical processes and outcomes, which may be influenced by variations in measurement practices and data availability, particularly for laboratory-based parameters. The precision of the reported estimates may also be influenced by the hierarchical structure of the data, with individuals clustered within healthcare facilities and states, as well as by incomplete data availability for certain indicators. These factors should be considered when interpreting variability across subgroups and geographic units. Given the cross-sectional nature of the analysis, causal inferences cannot be established. Fourth, no formal sample size calculation or power analysis was performed, as the study included all eligible records available within the institutional database during the study period. While 95% confidence intervals are presented to provide measures of precision, the study was not designed for formal hypothesis testing or inferential comparisons. Although this approach reflects real-world practice and supports external validity within the health system context, it may limit the ability to perform predefined inferential analyses typical of prospective study designs. Fifth, the absence of sufficiently standardized social variables, such as, educational attainment, socioeconomic position, or other equity stratifiers, limited formal assessment of equity-related disparities in the present analysis. Finally, as these findings represent early assessment from a recently implemented monitoring platform within an evolving health system under expansion, they should be interpreted within this implementation context.

In conclusion, early system-level assessment from Mexico’s emerging federalized public health system reveal heterogeneous progress toward Global Diabetes Compact 2030 targets, with blood pressure control approaching target levels, but persistent gaps in glycemic control and statin use. Closing implementation gaps in diabetes care is essential and will only be achieved through coordinated system-level interventions. Integrated monitoring platforms based on routinely collected clinical data offer a scalable approach to track progress, identify priority areas, and support continuous improvement in diabetes care delivery across diverse settings. These findings highlight key opportunities to strengthen diabetes care, particularly through improved comprehensive diabetes management and targeted strategies for high-risk groups.

## Data Availability

The data supporting the findings of this study are derived from routinely collected clinical records and are not publicly available because they may contain potentially identifiable individual-level information. A de-identified coded dataset may be made available upon reasonable request, subject to institutional approval and applicable privacy safeguards.
