# Supplementary figures and images for: Early assessment of diabetes care target attainment in Mexico’s federalized public health system: alignment with Global Diabetes Compact 2030
Source: Front Clin Diabetes Healthc. 2026 Jun 15;7:1837250. doi: 10.3389/fcdhc.2026.1837250 (PMC13310749; doi:10.3389/fcdhc.2026.1837250)

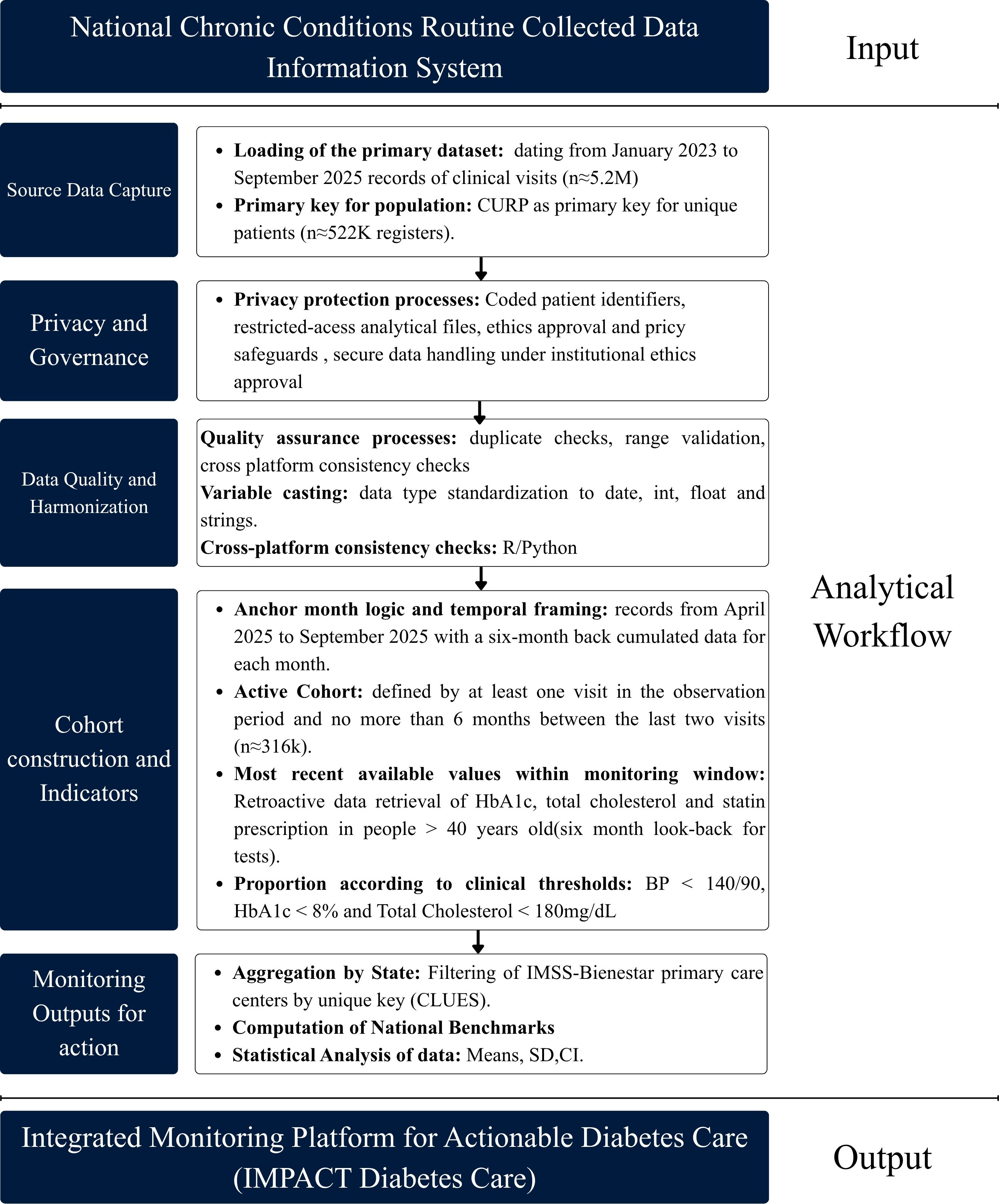

Supplement: Supplementary file 1 [file Image1.jpeg]

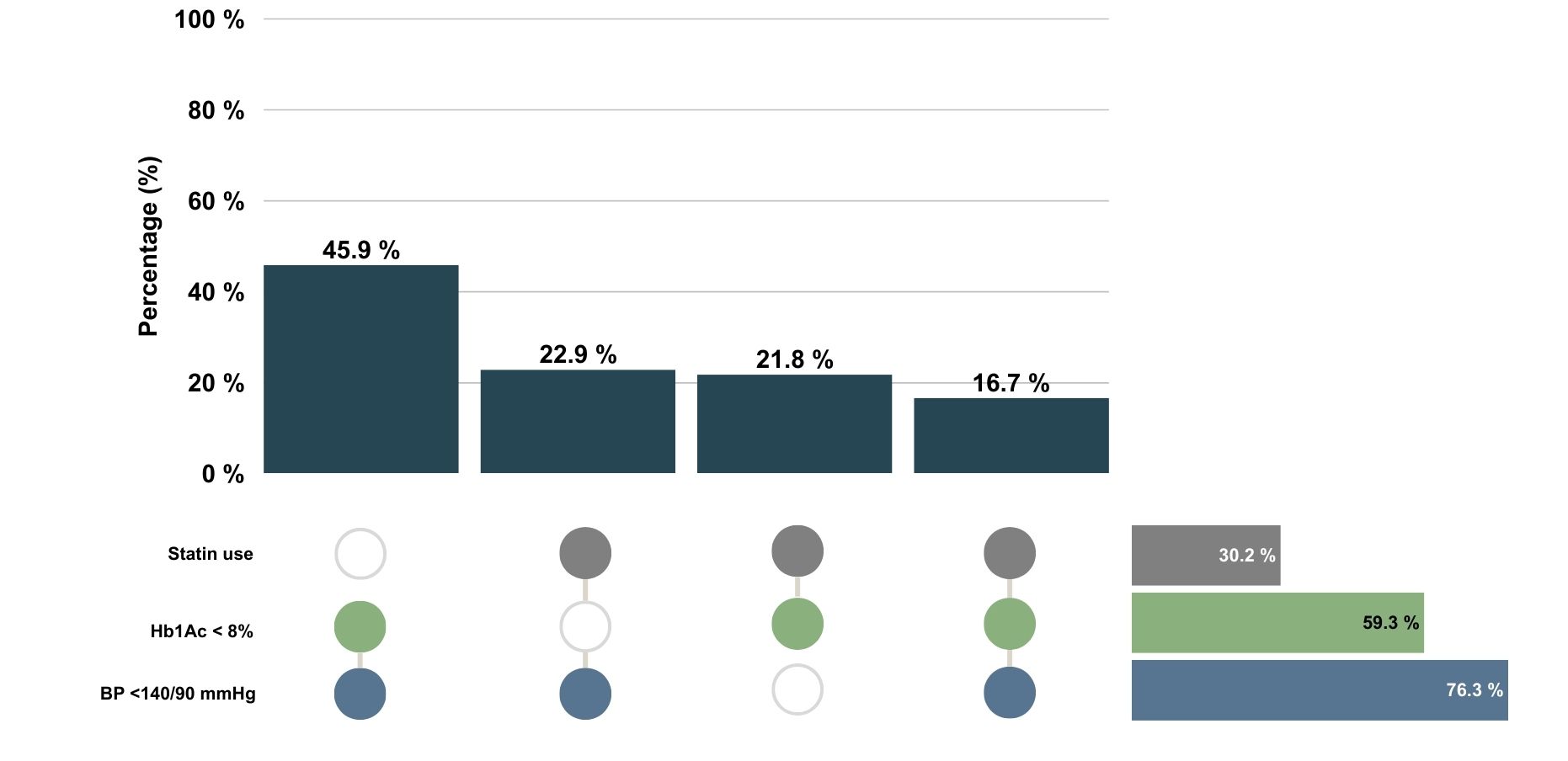

Supplement: Supplementary file 3 [file Image3.jpeg]
