# Supplementary material for: Early assessment of diabetes care target attainment in Mexico’s federalized public health system: alignment with Global Diabetes Compact 2030
Source: Front Clin Diabetes Healthc. 2026 Jun 15;7:1837250. doi: 10.3389/fcdhc.2026.1837250 (PMC13310749; doi:10.3389/fcdhc.2026.1837250)

**Supplementary Figure 2.** Flow diagram showing the derivation of the analytic sample from the total number of individuals with diabetes registered in the Sistema de Información en Crónicas (SIC) between January 2023 and September 2025. Individuals without a clinical encounter within the six months preceding the anchor month were excluded. The diagram also presents the number of individuals with available data for each key indicator, including HbA1c, cholesterol, and statin use. Estimates for each indicator were calculated among individuals with available data within the monitoring period.

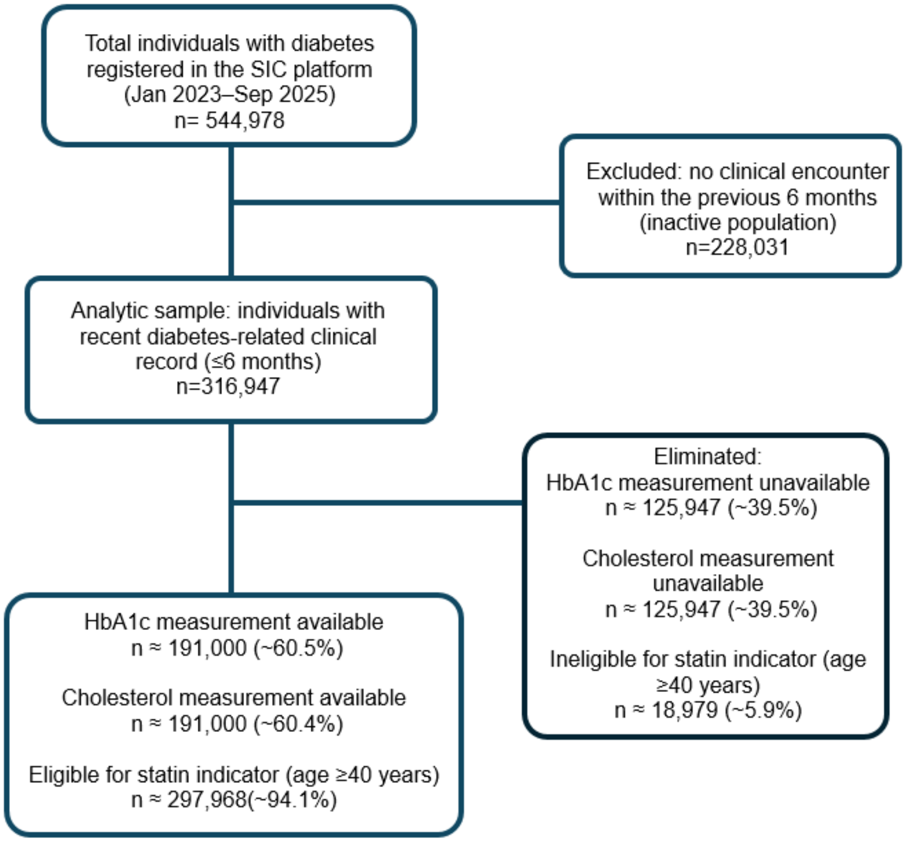

Supplement: Supplementary file 2 [file Image2.pdf]
