# Supplementary material for: Early assessment of diabetes care target attainment in Mexico’s federalized public health system: alignment with Global Diabetes Compact 2030
Source: Front Clin Diabetes Healthc. 2026 Jun 15;7:1837250. doi: 10.3389/fcdhc.2026.1837250 (PMC13310749; doi:10.3389/fcdhc.2026.1837250)
Supplement: Supplementary file 4 [file Table1.docx]

**Supplementary Table 1**. Characteristics of individuals with a recent record of diabetes-related care

| **Variable** | **Total n=316,947** | **95% CI** |
| --- | --- | --- |
| **Sex, n (%)** |  |  |
| Men | 89,481 (28.2) | 28.1 - 28.4 |
| Women | 227,466 (71.7) | 71.6 - 71.9 |
| **Age, n (%)** |  |  |
| <40 years | 18,979 (5.9) | 5.6 - 6.2 |
| 40-59 years | 139,996 (44.1) | 43.8 - 44.4 |
| ≥60 years | 157,972 (49.8) | 49.6 - 50.1 |
| **Duration of disease, n (%)** |  |  |
| <5 years | 117,253 (37) | 36.7 - 37.3 |
| 6-10 years | 73,402 (23.2) | 22.9 - 23.5 |
| 11-15 years | 59,108 (18.6) | 18.3 - 18.9 |
| >15 years | 67,184 (21.2) | 20.9 - 21.5 |
| BMI, kg/m^2^ | 28.3 (25.2 - 32) | 28.2 - 28.3 |
| **Distribution of BMI categories^‡^, n (%)** |  |  |
| <25 kg/m^2^ | 72,935 (23.1) | 23 - 23.3 |
| 25-30 kg/m^2^ | 123,941 (39.1) | 39 - 39.4 |
| >30 kg/m^2^ | 118,655 (37.4) | 37.2 - 37.6 |
| **Comorbidities, n (%)** |  |  |
| Hypertension | 153,291 (48.3) | 48 - 48.6 |
| Obesity (BMI) | 118,655 (37.4) | 37.1 - 37.7 |
| Obesity (waist circumference) | 277,061 (91.3) | 90.9 - 91.7 |
| Cardiovascular disease | 8,009 (2.5) | 2.4 - 2.6 |
| **HbA1c*****, %** | 7.94 ± 2.3 | 7.93 - 7.95 |
| **HbA1c in control*****, n (%)** |  |  |
| <7.0% | 81,837 (42.6) | 42.4 - 42.9 |
| <8.0% | 113,669 (59.2) | 58.9 - 59.4 |
| **SBP, mmHg** | 123.5 ± 18.8 | 123.4 - 123.6 |
| **DBP, mmHg** | 74.18 ± 10.5 | 74.14 - 74.21 |
| **BP in control, n (%)** |  |  |
| <140/90 mmHg | 241,496 (76.6) | 76.4 - 76.8 |
| <130/80 mmHg | 150,931 (47.9) | 47.7 - 48.2 |
| **Lipids** |  |  |
| Total cholesterol^µ^ | 188.3 ± 80.6 | 187 - 189.3 |
| Cholesterol <200 mg/dL^µ^, n (%) | 127,439 (66.5) | 66.2 - 66.8 |
| Cholesterol <180 mg/dL^µ^, n (%) | 96, 234 (50.2) | 49.9 - 50.5 |
| Statin prescription (>40 yrs) | 91,349 (30.6) | 30.3 - 30.9 |

BMI: body mass index; CKD: chronic kidney disease; eGFR: estimated glomerular filtration rate; HbA1c: glycated hemoglobin; BP: blood pressure; HDL: high-density lipoprotein; LDL: low-density lipoprotein. Continuous variables are presented as mean ± SD or median (p25-p75), as appropriate. **^‡^** Missing data <1%, *Available for 60.5% of individuals included in the analysis,^µ^available for 60.4% of individuals included in the analysis.

**Supplementary Table S2**. Characteristics of individuals with and without HbA1c test availability.

| **Variable** | **With test**  **Total n= 191,767** | **Without test**  **Total n= 125,180** | **p-value** |
| --- | --- | --- | --- |
| **Sex, n (%)** |  |  |  |
| Men | 52,258 (27.3) | 37,225(29.7) | <0.001 |
| Women | 139,509 (72.7) | 87,955 (70.3) |  |
| **Age, n (%)** |  |  |  |
| <40 years | 14,316 (7.5) | 10,594 (8.4) | <0.001 |
| 40-59 years | 98,061(52.5) | 63,780 (51.0) |  |
| ≥60 years | 74,720 (40.0) | 50,803 (40.6) |  |
| **Duration of disease, n (%)** |  |  |  |
| <5 years | 68,775 (35.9) | 48,483 (38.7) | <0.001 |
| 6-10 years | 44,672 (23.3) | 28,730 (23.0) |  |
| 11-15 years | 36,118( 18.8) | 22,988 (18.4) |  |
| >15 years | 42,202( 22.0) | 24,979 (20.0) |  |
| Hb1Ac control < 8%, n (%) | 113,661 (59.3) | N/A |  |
| BP <140/90 mmHg, n (%) | 148,101(77.2) | 93,405 (74.6) | <0.001 |

HbA1c: glycated hemoglobin; BP: blood pressure; N/A: No Applicable.

**Supplementary Table S3**. Characteristics of individuals with and without test availability

| **Variable** | **With test**  **Total n= 191,767** | **Without test**  **Total n= 125,180** | **p-value** |
| --- | --- | --- | --- |
| **Sex, n (%)** |  |  |  |
| Men | 51,939 (27.1) | 37,544 (29.9) | <0.001 |
| Women | 139,567 (72.9) | 87,897 (70.1) |  |
| **Age, n (%)** |  |  |  |
| <40 years | 14,197 (7.4) | 10,713 (8.5) | <0.001 |
| 40-59 years | 100,803 (52.6) | 63,707 (50.8) |  |
| ≥60 years | 76, 503 (39.9) | 51,020 (40.7) |  |
| **Duration of disease, n (%)** |  |  |  |
| <5 years | 69,122 (36.1) | 48,136 (38.4) | <0.001 |
| 6-10 years | 44,610 (23.3) | 23,037 (18.4) |  |
| 11-15 years | 36, 069 (18.8) | 25,476 (20.3) |  |
| >15 years | 41,705 (21.8) | 25,476 (20.3) |  |
| HbA1c control < 8%, n (%) | 89,172 (46.6) | 24,489 (19.5) | <0.001 |
| BP control <140/90 mmHg, n (%) | 147, 344(76.9) | 94,162 (75.1) | <0.001 |

HbA1c: glycated hemoglobin; BP: blood pressure; Continuous variables are presented as mean ± SD or median (p25-p75), as appropriate. **^‡^**

**Supplementary Table S4**. Attainment of three key diabetes care targets overall and by sex and age group.

| **Subgroup** | **HbA1c <8%** | | **BP <140/90** | | **Statin use** | | **All three met** | |
| --- | --- | --- | --- | --- | --- | --- | --- | --- |
|  | **%** | **95% CI** | **%** | **95% CI** | **%** | **95% CI** | **%** | **95% CI** |
| **Overall** | | | | | | | | |
| **Total** | 59.3 | 59.1–59.5 | 76.6 | 76.5–76.8 | 30.2 | 30.1–30.4 | 16.7 | 16.5–16.8 |
| **<40 years** | 48.2 | 47.2–49.2 | 87.5 | 86.9–88 | 22.6 | 21.9–23.2 | 11.8 | 11.1–12.5 |
| **40–59 years** | 53.0 | 52.7–53.4 | 80.6 | 80.3–80.8 | 30.3 | 30.1–30.6 | 15.8 | 15.6–16.1 |
| **≥60 years** | 64.8 | 64.5–65.1 | 72.7 | 72.5–72.9 | 30.8 | 30.6–31 | 17.7 | 17.5–17.9 |
| **Men** | | | | | | | | |
| **Total men** | 59.3 | 58.9-59.7 | 73.3 | 73.0–73.6 | 27.6 | 27.3–27.9 | 14.8 | 14.5–15.1 |
| **<40 years** | 44.2 | 42.2- 46.3 | 83.2 | 82.1–84.4 | 23.2 | 21.9–24.5 | 11.7 | 10.5–13.1 |
| **40–59 years** | 52 | 51.3- 52.7 | 75.9 | 75.4–76.3 | 27.5 | 27.0–28.0 | 13.5 | 13–14 |
| **≥60 years** | 65 | 64.4- 65.5 | 70.8 | 70.4–71.2 | 27.9 | 27.6–28.3 | 15.9 | 15.5–16.3 |
| **Women** | | | | | | | | |
| **Total women** | 59.3 | 59-59.5 | 78 | 77.8–78.1 | 31.2 | 31.1–31.4 | 17.3 | 17.1–17.5 |
| **<40 years** | 49.6 | 48.4-50.8 | 89 | 88.4–89.5 | 22.4 | 21.6–23.1 | 11.8 | 11.1–12.6 |
| **40–59 years** | 53.4 | 53-53.8 | 82.2 | 82–82.4 | 31.3 | 31.0–31.6 | 16.6 | 16.3–16.9 |
| **≥60 years** | 64.7 | 64.4-65.1 | 73.6 | 73.3–73.8 | 32.0 | 31.8–32.3 | 18.4 | 18.2–18.7 |

HbA1c: glycated hemoglobin; BP: blood pressure. Values are proportions (%) with 95% confidence intervals (CI).

**Supplementary Table S5**. Characteristics of individuals with a recent record of diabetes-related care and without a recent follow-up.

| **Variable** | **Recent follow-up**  **Total n=316,947** | **Without recent follow-up**  **Total n= 21,556** | **p-value** |
| --- | --- | --- | --- |
| **Sex, n (%)** |  |  |  |
| Men | 89,481 (28.2) | 6,803 (31.6) | <0.001 |
| Women | 227,466 (71.7) | 14,753 (68.4) |  |
| **Age, n (%)** |  |  |  |
| <40 years | 18,979 (5.9) | 1,992 (9.2) | <0.001 |
| 40-59 years | 139,996 (44.1) | 11,302 (52.4) |  |
| ≥60 years | 157,972 (49.8) | 8,260 (38.3) |  |
| **Duration of disease, n (%)** |  |  |  |
| <5 years | 117,253 (37) | 8,534 (39.6) | <0.001 |
| 6-10 years | 73,402 (23.2) | 4,832 (22.4) |  |
| 11-15 years | 59,108 (18.6) | 3,772(17.5) |  |
| >15 years | 67,184 (21.2) | 4,418 (20.5) |  |
| BMI, kg/m^2^ | 28.3 (25.2 - 32) | 28.38(25.1 - 32.2) | 0.96 |
| BMI, kg/m^2^ | 29.6 ± 14.3 | 29.85 ± 15.56 | 0.04 |
| **Distribution of BMI categories^‡^, n (%)** | | | |
| <25 kg/m^2^ | 72,935 (23) | 5,538(24.3) | <0.001 |
| 25-30 kg/m^2^ | 123,941 (39.1) | 8,126(37.7) |  |
| >30 kg/m^2^ | 118,655 (37.4) | 8,191(38) |  |
| **Comorbidities, n (%)** |  |  |  |
| Hypertension | 153,291 (48.3) | 9,619(44.6) | <0.001 |
| Obesity (BMI) | 118,655 (37.4) | 8,030(37.3) |  |
| Obesity (waist circumference; >80 cm in women and >90 cm in men) | 277,061 (91.3) | 17,382(80.6) |  |
| Cardiovascular disease | 8,009 (2.5) | 441(2.0) |  |
| **HbA1c*****, %** | 7.9 ± 2.3 | 8.16 ± 2.47 | <0.001 |
| **HbA1c in control*****, n (%)** |  |  |  |
| <7.0% | 81,837 (42.6) | 3,830(38.6) | <0.001 |
| <8.0% | 113,669 (59.2) | 5,450(54.9) | <0.001 |
| **SBP, mmHg** | 123.5 ± 18.8 | 124.9 ± 20.2 | <0.001 |
| **DBP, mmHg** | 74.1 ± 10.5 | 75.3 ± 11 | <0.001 |
| **BP in control, n (%)** |  |  |  |
| <140/90 mmHg | 241,496 (76.6) | 15,504(73.3) | <0.001 |
| <130/80 mmHg | 150,931 (47.9) | 9,270(43.8) | <0.001 |
| **Lipids** |  |  |  |
| Total cholesterol^µ^ | 188.3 ± 80.6 | 190.45 ± 95.2 | 0.22 |
| Cholesterol <200 mg/dL^µ^, n (%) | 127,439 (66.5) | 5,619 (65.4) | 0.58 |
| Cholesterol <180 mg/dL^µ^, n (%) | 96,234 (50.2) | 4,296 (50.1) | 0.58 |
| Statin prescription (>40 yrs) | 91,349 (30.6) | 5,695 (29.1) | <0.001 |

BMI: body mass index; HbA1c: glycated hemoglobin; BP: blood pressure;Continuous variables are presented as mean ± SD or median (p25-p75), as appropriate. **^‡^** Missing data <1%, *Data availability for the analyzed cohort: 60.5% (active) and 46.1% (inactive), ^µ^Data availability for the analyzed cohort 60.4% (active) and 46.1% (inactive).
